# Poly[(μ_6_-6-oxidopyridinium-2-carboxyl­ato)caesium]

**DOI:** 10.1107/S1600536811031874

**Published:** 2011-08-17

**Authors:** Sung Kwon Kang, Yong Suk Shim

**Affiliations:** a Department of Chemistry, Chungnam National University, Daejeon 305-764, Republic of Korea

## Abstract

The asymmetric unit of the polymeric title salt, [Cs(C_6_H_4_NO_3_)]_*n*_, comprises a Cs^+^ cation and a 6-oxidopyridinium-2-carboxyl­ate anion. The Cs^+^ cation is six-coordinated by O atoms derived from two oxido and four carboxyl­ate O atoms; each O atom in the anion bridges two Cs^+^ cations. In the crystal, inter­molecular N—H⋯O hydrogen bonding is present and contributes to the stability of the three-dimensional network generated by the bridging O atoms.

## Related literature

For general background to pyridine carb­oxy­lic complexes, see: Kang (2011 ▶); Lee & Kang (2010 ▶); Hong *et al.* (2008 ▶). For the Cs—O bond lengths in caesium aryl­oxide complexes, see: Ungaro *et al.* (1994 ▶); Clark *et al.* (1998 ▶); Weinert *et al.* (2003 ▶). 
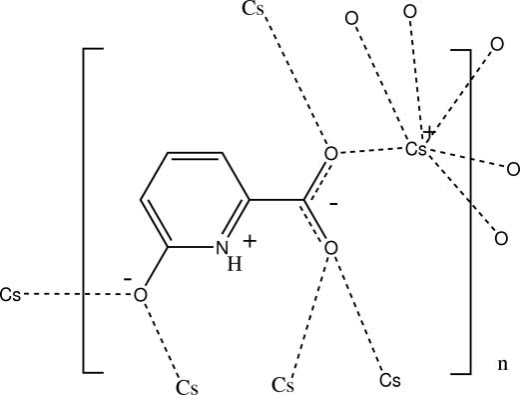


## Experimental

### 

#### Crystal data

[Cs(C_6_H_4_NO_3_)]
*M* *_r_* = 271.01
Monoclinic, 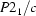

*a* = 8.1746 (3) Å
*b* = 7.5513 (2) Å
*c* = 12.3843 (4) Å
β = 91.889 (1)°
*V* = 764.05 (4) Å^3^
*Z* = 4
Mo *K*α radiation
μ = 4.8 mm^−1^
*T* = 296 K
0.10 × 0.07 × 0.06 mm

#### Data collection

Bruker SMART CCD area-detector diffractometer
Absorption correction: multi-scan (*SADABS*; Bruker, 2002 ▶) *T*_min_ = 0.654, *T*_max_ = 0.745
6897 measured reflections
1822 independent reflections
1592 reflections with *I* > 2σ(*I*)
*R*_int_ = 0.072

#### Refinement

*R*[*F* ^2^ > 2σ(*F* ^2^)] = 0.028
*wR*(*F* ^2^) = 0.072
*S* = 1.00
1822 reflections
104 parameters
H atoms treated by a mixture of independent and constrained refinement
Δρ_max_ = 1.19 e Å^−3^
Δρ_min_ = −1.14 e Å^−3^

### 

Data collection: *SMART* (Bruker, 2002 ▶); cell refinement: *SAINT* (Bruker, 2002 ▶); data reduction: *SAINT*; program(s) used to solve structure: *SHELXS97* (Sheldrick, 2008 ▶); program(s) used to refine structure: *SHELXL97* (Sheldrick, 2008 ▶); molecular graphics: *ORTEP-3 for Windows* (Farrugia, 1997 ▶) and *DIAMOND* (Brandenburg, 2010 ▶)’; software used to prepare material for publication: *WinGX* (Farrugia, 1999 ▶).

## Supplementary Material

Crystal structure: contains datablock(s) global, I. DOI: 10.1107/S1600536811031874/tk2776sup1.cif

Structure factors: contains datablock(s) I. DOI: 10.1107/S1600536811031874/tk2776Isup2.hkl

Additional supplementary materials:  crystallographic information; 3D view; checkCIF report

## Figures and Tables

**Table 1:** Selected bond lengths (Å)

Cs1—O9^i^	2.938 (2)
Cs1—O10^ii^	2.991 (3)
Cs1—O9	3.070 (3)
Cs1—O10^iii^	3.105 (3)
Cs1—O11^iv^	3.147 (2)
Cs1—O11^v^	3.317 (2)

Symmetry codes: (i) 
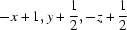
; (ii) 
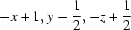
; (iii) 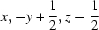; (iv) 
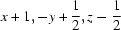
; (v) 
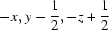
.

**Table 2:** Hydrogen-bond geometry (Å, °)

*D*—H⋯*A*	*D*—H	H⋯*A*	*D*⋯*A*	*D*—H⋯*A*
N2—H2⋯O11^vi^	0.78 (3)	2.15 (3)	2.915 (4)	168 (3)

Symmetry code: (vi) 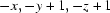.

**Fig. 1.**
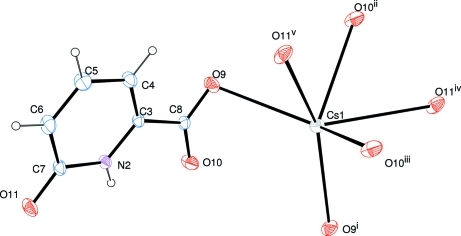
Molecular structure of (l), showing the atom-numbering scheme and 20% probability ellipsoids. [Symmetry code: (i) -x + 1, y + 1/2, -z + 1/2; (ii) -x + 1, y - 1/2, -z + 1/2; (iii) x, -y + 1/2, z - 1/2; (iv) x + 1, -y + 1/2, z - 1/2; (v) -x, y - 1/2, -z + 1/2].

**Fig. 2.**
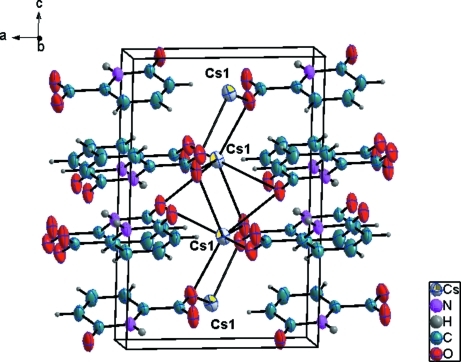
The three-dimensional framework of (I).

## supplementary crystallographic information

### Comment

During studies of lanthanide complexes of picolinic acid and their derivatives due to their interesting photoluminescent properties (Kang, 2011; Lee & Kang, 2010; Hong *et al.*, 2008), the title compound was obtained as a side-product.

The asymmetric unit of the title compound, [Cs(C_6_H_4_NO_3_)]_n_, comprises a Cs^+^ cation and a carboxylatooxidopyridinium anion. The Cs^+^ cation is coordinated to the two oxide O atoms and four carboxylate-O atoms (Fig. 1). The Cs—O bond distances lie within the range 2.938 (2) - 3.317 (2) Å (Table 1). The observed Cs—O distances are a little longer than those observed in caesium picrate complexes and caesium phenoxide complexes (Ungaro *et al.*, 1994: Clark *et al.*, 1998; Weinert *et al.*, 2003). The dihedral angle between the pyridine ring and the carboxylate group is 6.95 (19) °. In the crystal structure, the Cs atoms are linked by O atoms of the anionic ligands to form a three-dimensional network (Fig. 2) with additional stability provided by intermolecular N—H···O hydrogen bonding (Table 2).

### Experimental

Europium trichloride solution was prepared by dissolving EuCl_3_ 6H_2_O (0.37 g, 1.0 mmol; Aldrich) in absolute ethanol (20 ml) at room temperature with stirring. The ligand solution was prepared by dissolving 6-hydroxypicolinic acid (0.56 g, 4.0 mmol; Aldrich) in absolute ethanol (30 ml) at room temperature. The pH of the ligand solution was adjusted to about 6 with 2 N CsOH solution. The Eu solution was added drop wise and slowly to the ligand solution. The reaction mixture was stirred for 2 h at room temperature. Colourless crystals of (I) were obtained at room temperature over a period of a few weeks. The complex was recrystallized from distilled water.

### Refinement

The N—H atom was located in a difference Fourier map and refined freely. The remaining H atoms were positioned geometrically and refined using a riding model with C—H = 0.93 Å, and with *U*_iso_(H) = 1.2*U*_eq_ (C). The maximum and minimum residual electron density peaks of 1.19 and -1.14 e Å^-3^, respectively, were located 0.83 Å and 0.71 Å from the Cs1 atom, respectively.

### Crystal data

**Table:**

[Cs(C_6_H_4_NO_3_)]	*F*(000) = 504
*M**_r_* = 271.01	*D*_x_ = 2.356 Mg m^−^^3^
Monoclinic, *P*2_1_/*c*	Mo *K*α radiation, λ = 0.71073 Å
Hall symbol: -P 2ybc	Cell parameters from 3429 reflections
*a* = 8.1746 (3) Å	θ = 2.5–28.3°
*b* = 7.5513 (2) Å	µ = 4.8 mm^−^^1^
*c* = 12.3843 (4) Å	*T* = 296 K
β = 91.889 (1)°	Block, colourless
*V* = 764.05 (4) Å^3^	0.1 × 0.07 × 0.06 mm
*Z* = 4	

### Data collection

**Table:**

Bruker SMART CCD area-detector diffractometer	1592 reflections with *I* > 2σ(*I*)
φ and ω scans	*R*_int_ = 0.072
Absorption correction: multi-scan (*SADABS*; Bruker, 2002)	θ_max_ = 28.3°, θ_min_ = 2.5°
*T*_min_ = 0.654, *T*_max_ = 0.745	*h* = −3→10
6897 measured reflections	*k* = −10→7
1822 independent reflections	*l* = −15→15

### Refinement

**Table:**

Refinement on *F*^2^	0 restraints
Least-squares matrix: full	H atoms treated by a mixture of independent and constrained refinement
*R*[*F*^2^ > 2σ(*F*^2^)] = 0.028	*w* = 1/[σ^2^(*F*_o_^2^) + (0.0401*P*)^2^] where *P* = (*F*_o_^2^ + 2*F*_c_^2^)/3
*wR*(*F*^2^) = 0.072	(Δ/σ)_max_ = 0.001
*S* = 1.00	Δρ_max_ = 1.19 e Å^−^^3^
1822 reflections	Δρ_min_ = −1.14 e Å^−^^3^
104 parameters	

### Special details

**Table:**

Geometry. All e.s.d.'s (except the e.s.d. in the dihedral angle between two l.s. planes) are estimated using the full covariance matrix. The cell e.s.d.'s are taken into account individually in the estimation of e.s.d.'s in distances, angles and torsion angles; correlations between e.s.d.'s in cell parameters are only used when they are defined by crystal symmetry. An approximate (isotropic) treatment of cell e.s.d.'s is used for estimating e.s.d.'s involving l.s. planes.

### Fractional atomic coordinates and isotropic or equivalent isotropic displacement parameters (Å^2^)

**Table:**

	*x*	*y*	*z*	*U*_iso_*/*U*_eq_	
Cs1	0.51201 (2)	0.14792 (3)	0.127051 (18)	0.04196 (11)	
N2	0.0320 (3)	0.2743 (4)	0.4216 (2)	0.0304 (5)	
H2	0.088 (4)	0.348 (4)	0.446 (3)	0.028 (9)*	
C3	0.1062 (3)	0.1326 (4)	0.3746 (3)	0.0291 (6)	
C4	0.0159 (4)	−0.0019 (5)	0.3335 (3)	0.0402 (8)	
H4	0.0655	−0.0992	0.3023	0.048*	
C5	−0.1567 (4)	0.0085 (5)	0.3391 (3)	0.0474 (9)	
H5	−0.221	−0.0826	0.3102	0.057*	
C6	−0.2296 (4)	0.1484 (5)	0.3857 (3)	0.0444 (9)	
H6	−0.343	0.152	0.3888	0.053*	
C7	−0.1347 (3)	0.2903 (5)	0.4302 (3)	0.0334 (7)	
C8	0.2926 (3)	0.1403 (4)	0.3700 (3)	0.0298 (6)	
O9	0.3603 (3)	0.0066 (3)	0.3335 (2)	0.0487 (6)	
O10	0.3588 (2)	0.2803 (4)	0.3984 (2)	0.0536 (7)	
O11	−0.1942 (2)	0.4235 (3)	0.4750 (2)	0.0498 (7)	

### Atomic displacement parameters (Å^2^)

**Table:**

	*U*^11^	*U*^22^	*U*^33^	*U*^12^	*U*^13^	*U*^23^
Cs1	0.03869 (13)	0.03507 (16)	0.05252 (19)	−0.00018 (7)	0.00759 (9)	−0.00010 (9)
N2	0.0203 (10)	0.0308 (14)	0.0402 (16)	−0.0019 (10)	0.0026 (9)	−0.0062 (12)
C3	0.0234 (12)	0.0316 (16)	0.0324 (17)	0.0012 (10)	0.0034 (10)	0.0006 (12)
C4	0.0313 (14)	0.0409 (19)	0.049 (2)	−0.0026 (12)	0.0044 (12)	−0.0145 (16)
C5	0.0316 (15)	0.049 (2)	0.061 (3)	−0.0137 (14)	−0.0003 (14)	−0.0201 (18)
C6	0.0216 (13)	0.056 (2)	0.055 (2)	−0.0055 (12)	0.0003 (13)	−0.0125 (17)
C7	0.0209 (11)	0.0393 (17)	0.0401 (19)	0.0013 (11)	0.0030 (10)	−0.0036 (15)
C8	0.0226 (12)	0.0325 (17)	0.0346 (18)	0.0020 (10)	0.0045 (10)	0.0017 (12)
O9	0.0321 (11)	0.0421 (14)	0.0725 (19)	0.0074 (10)	0.0112 (10)	−0.0090 (13)
O10	0.0235 (9)	0.0383 (14)	0.099 (2)	−0.0025 (9)	0.0070 (11)	−0.0189 (15)
O11	0.0244 (10)	0.0483 (15)	0.0772 (19)	0.0022 (10)	0.0070 (10)	−0.0231 (14)

### Geometric parameters (Å, °)

**Table:**

Cs1—O9^i^	2.938 (2)	C5—C6	1.352 (5)
Cs1—O10^ii^	2.991 (3)	C5—H5	0.93
Cs1—O9	3.070 (3)	C6—C7	1.423 (4)
Cs1—O10^iii^	3.105 (3)	C6—H6	0.93
Cs1—O11^iv^	3.147 (2)	C7—O11	1.254 (4)
Cs1—O11^v^	3.317 (2)	C8—O10	1.234 (4)
N2—C3	1.370 (4)	C8—O9	1.244 (4)
N2—C7	1.376 (3)	O9—Cs1^ii^	2.938 (2)
N2—H2	0.78 (3)	O10—Cs1^i^	2.991 (3)
C3—C4	1.345 (4)	O10—Cs1^vi^	3.105 (3)
C3—C8	1.527 (4)	O11—Cs1^vii^	3.147 (2)
C4—C5	1.418 (4)	O11—Cs1^viii^	3.317 (2)
C4—H4	0.93		
			
O9^i^—Cs1—O10^ii^	138.47 (6)	C3—N2—C7	123.8 (3)
O9^i^—Cs1—O9	109.46 (5)	C3—N2—H2	118 (3)
O10^ii^—Cs1—O9	85.35 (7)	C7—N2—H2	119 (3)
O9^i^—Cs1—O10^iii^	96.96 (7)	C4—C3—N2	120.3 (3)
O10^ii^—Cs1—O10^iii^	101.48 (6)	C4—C3—C8	123.4 (3)
O9—Cs1—O10^iii^	131.16 (6)	N2—C3—C8	116.3 (2)
O9^i^—Cs1—O11^iv^	89.05 (7)	C3—C4—C5	118.3 (3)
O10^ii^—Cs1—O11^iv^	59.91 (6)	C3—C4—H4	120.8
O9—Cs1—O11^iv^	140.90 (6)	C5—C4—H4	120.8
O10^iii^—Cs1—O11^iv^	77.13 (6)	C6—C5—C4	121.1 (3)
O9^i^—Cs1—O11^v^	143.52 (6)	C6—C5—H5	119.4
O10^ii^—Cs1—O11^v^	76.13 (6)	C4—C5—H5	119.4
O9—Cs1—O11^v^	78.86 (6)	C5—C6—C7	120.8 (3)
O10^iii^—Cs1—O11^v^	56.95 (6)	C5—C6—H6	119.6
O11^iv^—Cs1—O11^v^	106.75 (4)	C7—C6—H6	119.6
O9^i^—Cs1—O10	74.53 (6)	O11—C7—N2	120.3 (3)
O10^ii^—Cs1—O10	118.05 (6)	O11—C7—C6	124.1 (3)
O9—Cs1—O10	36.14 (6)	N2—C7—C6	115.6 (3)
O10^iii^—Cs1—O10	129.22 (5)	O10—C8—O9	127.1 (3)
O11^iv^—Cs1—O10	149.72 (6)	O10—C8—C3	116.8 (3)
O11^v^—Cs1—O10	101.34 (6)	O9—C8—C3	116.0 (3)
O9^i^—Cs1—C7^iv^	74.04 (7)	Cs1^ii^—O9—Cs1	107.94 (7)
O10^ii^—Cs1—C7^iv^	76.81 (7)	Cs1^i^—O10—Cs1^vi^	78.52 (6)
O9—Cs1—C7^iv^	153.81 (6)	Cs1^i^—O10—Cs1	91.33 (7)
O10^iii^—Cs1—C7^iv^	72.02 (6)	Cs1^vi^—O10—Cs1	136.48 (6)
O11^iv^—Cs1—C7^iv^	16.91 (7)	Cs1^vii^—O11—Cs1^viii^	73.25 (4)
O11^v^—Cs1—C7^iv^	114.40 (7)		

Symmetry codes: (i) −*x*+1, *y*+1/2, −*z*+1/2; (ii) −*x*+1, *y*−1/2, −*z*+1/2; (iii) *x*, −*y*+1/2, *z*−1/2; (iv) *x*+1, −*y*+1/2, *z*−1/2; (v) −*x*, *y*−1/2, −*z*+1/2; (vi) *x*, −*y*+1/2, *z*+1/2; (vii) *x*−1, −*y*+1/2, *z*+1/2; (viii) −*x*, *y*+1/2, −*z*+1/2.

### Hydrogen-bond geometry (Å, °)

**Table:**

*D*—H···*A*	*D*—H	H···*A*	*D*···*A*	*D*—H···*A*
N2—H2···O11^ix^	0.78 (3)	2.15 (3)	2.915 (4)	168 (3)

Symmetry codes: (ix) −*x*, −*y*+1, −*z*+1.
